# Dermoscopy of Melanoma According to Age Groups: A Retrospective Monocentric Study on 285 Patients

**DOI:** 10.3390/cancers17152597

**Published:** 2025-08-07

**Authors:** Francesco Cavallo, Umberto Santaniello, Elisa Bin, Gabriele Roccuzzo, Silvia Giordano, Andrea Agostini, Martina Merli, Paolo Fava, Pietro Quaglino, Simone Ribero, Paolo Broganelli

**Affiliations:** 1 Department of Medical Sciences, Section of Dermatology, University of Turin, 10124 Turin, Italy; umberto.santaniello@unito.it (U.S.); elisa.bin@edu.unito.it (E.B.); gabriele.roccuzzo@unito.it (G.R.); si.giordano@unito.it (S.G.); paolo.fava@unito.it (P.F.); pietro.quaglino@unito.it (P.Q.); simone.ribero@unito.it (S.R.); 2 Dermatology and Venereology, Mauriziano Umberto Hospital, 10128 Turin, Italy; aagostini@mauriziano.it (A.A.); mmerli@mauriziano.it (M.M.); pbroganelli@mauriziano.it (P.B.)

**Keywords:** melanoma, dermoscopy, skin cancer, dermoscopic pattern, Breslow thickness, early diagnosis

## Abstract

**Background/Objectives:** Melanoma is an aggressive skin cancer with increasing incidence worldwide. Dermoscopy has revolutionized early melanoma detection, but most studies have focused on the general adult population. This study aims to analyze dermoscopic and histological differences in melanoma across age groups, evaluating whether specific patterns vary between younger and older patients. **Methods:** This retrospective study included 285 histopathological confirmed melanomas diagnosed at the Dermatology Clinic of the University of Turin between November 2021 and April 2024. Patients were stratified by age (<40 vs. ≥40 years), and statistical analyses (Chi-square, logistic regression) assessed differences in dermoscopic, histopathological, and anatomical parameters. **Results**: Younger patients showed a higher prevalence of growth-related features (e.g., pseudopods, OR = 5.43; asymmetric globules, OR = 2.33) and a thicker Breslow index (mean = 1.05 mm). Older patients exhibited more regression-associated signs (scar-like depigmentation, OR = 0.15; peppering, OR = 0.39), greater lesion size, and solar elastosis. Dermoscopic regression significantly predicted histological regression, with age-stratified analysis revealing peppering as a predictor in younger patients (*p* = 0.015) and scar-like depigmentation in older ones (*p* = 0.012). **Conclusions**: Melanoma exhibits distinct dermoscopic features depending on patient age, with growth-associated patterns being more common in younger individuals and regressive patterns predominating in older patients. These findings highlight the importance of age-specific diagnostic considerations in melanoma detection, potentially improving early diagnosis and patient outcomes.

## Simple Summary

Melanoma is an aggressive skin cancer with a prognosis strongly depending on early detection. Dermoscopy has improved diagnostic accuracy, yet most studies focus on patients over 40, leaving dermoscopic features in younger individuals less explored. This study analyzed 285 melanomas across different age groups to assess variations in dermoscopic and histological features. Younger patients (<40 years) more frequently showed growth-related dermoscopic structures such as atypical globules and pseudopods and had a significantly higher mean Breslow thickness. In contrast, older patients (≥40 years) more often presented regression-related features, including scar-like depigmentation, peppering, larger lesion size, and solar elastosis. Dermoscopic signs of regression were predictive of histological regression, but the association varied by age: scar-like depigmentation was predictive only in older patients, whereas peppering remained significant in younger ones. These findings emphasize the role of age in the clinical assessment of melanoma and support a tailored dermoscopic interpretation to improve early diagnosis and risk stratification.

## 1. Introduction

Malignant melanoma (MM) is an aggressive type of skin cancer, and its incidence has been steadily increasing over the past few decades, especially among populations with fair skin. This rise can be attributed in part to greater exposure to ultraviolet light and changes in lifestyle habits. Epidemiological studies establish that incidence of MM in young adults has tripled since the 1960s, showing a gender-specific increase of over four times in males and more than eight times in females. MM is responsible for a significant proportion of skin cancer-related mortality worldwide [1,2,3]. Early detection of MM is critical for improving patient outcomes; indeed, prognosis is heavily influenced by clinicopathological factors such as Breslow thickness, ulceration and lymph-node involvement, highlighting the importance of prompt and accurate diagnosis [4]. Dermoscopy has dramatically changed the diagnosis of MM by improving the capability of the clinician to discern between benign and malignant lesions [5,6,7]. This cost-effective noninvasive technique provides a detailed visual representation of subclinical structures that are not visible to the naked eye, significantly improving diagnostic accuracy. The introduction of digital dermoscopy and videodermoscopy has further refined lesion monitoring in high-risk patients, allowing for a better identification of suspicious changes over time. However, most studies about dermoscopic diagnosis of MM have focused on the general adult population, particularly individuals over 40 years of age [8,9,10]. Consequently, there is limited evidence regarding whether current dermoscopic criteria are applicable across different age groups or if distinct age-related variations exist in melanoma morphology. Additionally, the biological behavior of MM varies with age: younger patients often exhibit neoplasm with unique molecular profiles and growth patterns. Recognizing these differences is essential to improving dermoscopic assessment and ensuring an early diagnosis of MM. This retrospective monocentric study aims to analyze the dermoscopic features of melanoma through different age groups by examining a cohort of 283 patients. Our objective is to evaluate the distribution of validated melanoma-specific dermoscopic criteria in younger versus older individuals, identifying potential differences that could improve diagnostic accuracy. By systematically assessing these patterns, we aim to provide a comprehensive overview that bridges the existing knowledge gap and refines the application of dermoscopy in melanoma detection. With the incidence of MM on the rise, mainly among younger patients, it is crucial to adapt diagnostic tools to consider age-related variations. This study represents a step toward optimizing dermoscopic evaluation by incorporating age as a significant variable in MM assessment. By highlighting these differences, our purpose is to improve early detection strategies and enhance patient outcomes, ultimately contributing to a more precise and effective approach to MM diagnosis.

## 2. Materials and Methods

We retrospectively reviewed 285 clinical and dermoscopic images of histologically confirmed MM from patient who attended the videodermatoscopy service at the Dermatologic Clinic of the Turin University Hospital, Italy, between November 2021 and April 2024. Patient inclusion criteria were the availability of a complete videodermatoscopy dataset enabling the assessment of melanoma specific dermoscopic patterns; the presence of a clinical image allowing for the evaluation of the location and distribution of the lesion; and the presence of a complete pathological report documenting the diagnosis of MM according to the TNM staging system/AJCC 8^th^ edition classification [11]. Exclusion criteria were the absence or insufficient quality of a clinical image; absence or insufficient quality of a dermoscopic image; and absence of a pathological report or MM located in anatomical site preventing dermoscopic examination. The binary age stratification (<40 vs. ≥40 years) was adopted based on epidemiological data demonstrating marked differences in melanoma incidence, tumor characteristics, and prognosis across these groups [12,13,14,15]. All patient data was obtained from the hospital’s database and subsequently archived within an internal computerized database, and the following data were extracted: age at MM diagnosis, sex, anatomical site of the lesion (categorized as face, scalp, anterior trunk, posterior trunk, upper limbs, lower, limbs, palmoplantar region, and other sites). Melanoma subtypes were classified according the 2018 WHO classification [16], including superficial spreading melanoma (SSM), lentigo maligna melanoma (LMM), nodular melanoma (NM), spitz melanoma (SM), acral lentiginous melanoma (ALM), mucosal melanoma, melanoma arising in congenital nevus, melanoma arising in blue nevus, and uveal melanoma. MM invasiveness was assessed according AJCC 8^th^ edition and categorized as pTis, pT1, pT2, pT3, or pT4 [11]. The following histopathological variables, as documented in the pathology reports, were collected and included in the study analysis: mitotic rate, ulceration, regression, and solar elastosis. The size of the MM was defined as the largest among the horizontal dimensions documented in the histopathological report. Each MM was evaluated for the presence of validated melanoma specific dermoscopic features such as abrupt ending of pigment pattern, atypical pigment network, atypical blotches, structureless areas, gray-blue veil, scar-like depigmentation, peppering, atypical dots/globules, pseudopods, radial streaks, polymorphous vessel, white shiny lines, angulated lines, negative pigment network, prominent skin markings, presence of gray color, and pattern or color asymmetry [8,17,18]. For each lesion, the predominant dermoscopic pattern was classified as globular, reticular, homogeneous, multicomponent, and non-specific (featureless or structureless or feature-poor lesions). Dermoscopic examination was performed using either Fotofinder Medicam 1000 (FotoFinder Systems GmbH, Bad Birnbach, Germany) or Vidix 4.0 (Canfield Scientific Inc., Parsippany, NJ, USA); Images were acquired under polarized light at a 10× magnification. Descriptive analysis evaluated frequencies, means, medians, standard deviations, and minimum and maximum values of each parameter, divided by age, sex, and anatomical site. Inferential analysis was performed using chi-square test to compare categorical variables. Fisher’s exact test was applied when expected frequencies were lower than 5. Univariate Logistic regression was used to estimate odds ratio (OR) and assess the strength of associations. Mann–Whitney U test was used to compare Breslow thickness and largest horizontal dimension means between young and elderly in the case of non-normal distribution. All statistical analyses were performed with STATA 17 software (StataCorp LLC, College Station, TX, USA).

## 3. Results

### 3.1. Population Charateristics

#### 3.1.1. General Population

A total of 285 MM from 285 patients were included in the study (mean age 61.3-year, median age 62 years, SD 15.65 years). Mean Breslow thickness was 0.49 mm (median 0 mm, SD 1.29 mm). Of these patients, 156 (54.74%) were male and 129 (45.26%) were female. The distribution of MM anatomical sites is summarized in Table 1.

#### 3.1.2. Population Under 40 Years of Age

The number of patients under 40 years of age was 36 (mean age 33.31 years, median 34.5 years, SD 7.12 years). Among these, 14 (38.89%) patients were male and 22 (61.11%) were female. Regarding Breslow thickness, the mean value was 1.05 mm (median 0.4 mm, SD 2.345 mm). The distribution of MM sites is summarized in Table 2.

#### 3.1.3. Population Aged 40 Years or Older

The number of patients aged 40 years or older was 249 (mean age 65.36 years, median age 65 years, SD 11.96 years). Of these, 107 (42.97%) were female and 142 (57.03%) were female. The mean Breslow thickness was 0.41 mm (median 0 mm, SD 1.034 mm). The distribution of MM location in patients aged 40 years or older is summarized in Table 3.

### 3.2. Breslow Thickness

**Figure 1:**
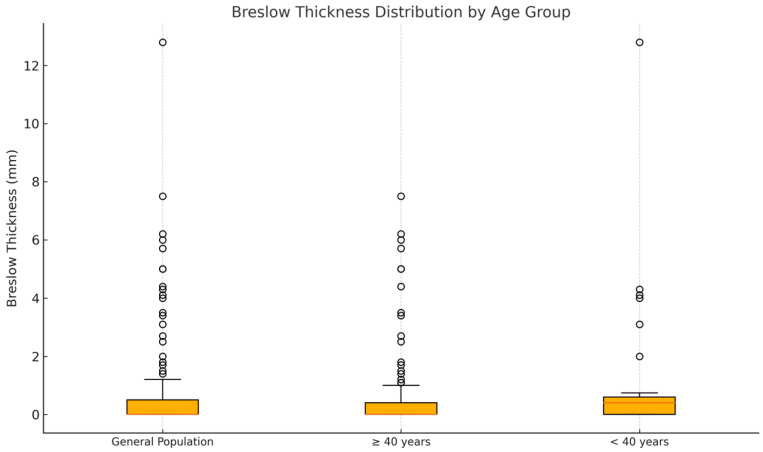
Distribution of Breslow thickness. *Y*-axis: Breslow thickness (mm). *X*-axis: patient groups (from left to right: all, ≥40 years old, <40 years old). The orange line within each box plot represents the median value of the distribution.

In our cohort, a statistically significant difference in Breslow thickness was observed between patients younger than 40 years and those aged 40 years or older, with the younger subgroup presenting a significantly higher Breslow thickness at diagnosis (Figure 1, *p* = 0.011, Mann–Whitney U test). Analysis of Breslow thickness showed that melanoma in situ was present in 161 out of 285 cases, representing 56.49% of the overall population. However, the frequency of melanoma in situ was notably lower in patients under 40 years, at 38.89% (14/36), compared to 59.04% (147/249) in patients aged 40 years or older. The mean Breslow thickness for the entire cohort was 0.49 mm with a median of 0.0 mm, ranging from 0.0 to 12.8 mm. When stratified by age, patients younger than 40 years had a mean Breslow thickness of 1.05 mm and a median of 0.40 mm, with a range from 0.0 to 12.8 mm, whereas patients aged 40 years or older had a mean Breslow thickness of 0.41 mm and a median of 0.0 mm, ranging from 0.0 to 7.5 mm. These results indicate that younger patients tend to present with thicker melanomas at diagnosis compared to older patients. 

### 3.3. Analysis of Difference in Size (Largest Horizontal Dimension)

**Figure 2:**
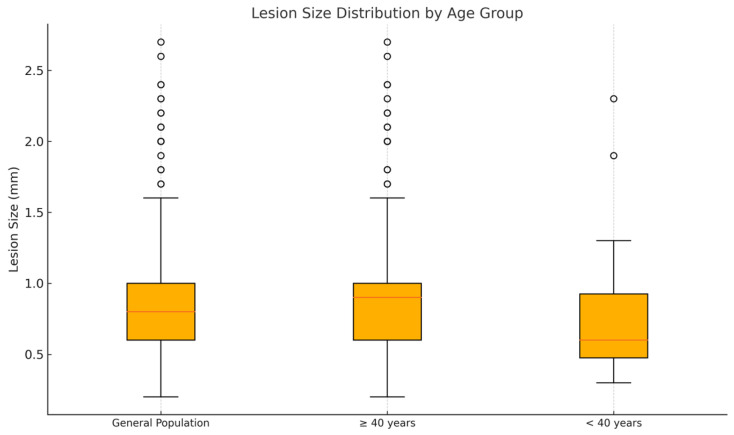
Distribution of size. *Y*-axis: largest horizontal dimension (mm). *X*-axis: patient groups (from left to right: all, ≥40 years old, <40 years old). The orange line within each box plot represents the median value of the distribution.

A comparative analysis of lesion size between the two age groups revealed a statistically significant difference, with patients aged 40 years and older presenting larger surface dimensions at diagnosis compared to those under 40 years (Figure 2, *p* = 0.0029, Mann–Whitney U test). The overall mean lesion size in the cohort was 0.88 cm, with a median of 0.8 cm and a range from 0.2 to 2.7 cm. When stratified by age, patients younger than 40 had a mean lesion size of 0.73 cm (median 0.6 cm, range 0.3–2.3 cm), whereas those aged 40 and above had a mean lesion size of 0.91 cm (median 0.9 cm, range 0.2–2.7 cm), indicating that melanomas tend to exhibit greater horizontal dimensions at diagnosis in the older patients’ group.

### 3.4. Crude (Unadjusted) Odds Ratios of Dermoscopic Parameters Beteween the Age Groups

The summary of the crude (unadjusted) odds ratios, 95% confidence intervals, and *p*-value results for the clinical, dermoscopic, and epidemiologic characteristics of the melanoma patients in the individual age groups are presented in Table 4.

A comparative analysis of dermoscopic features between patients younger and older than 40 years revealed several statistically significant differences. Pseudopods were observed in 33 cases (11.6% overall), with a significantly higher frequency in patients under 40 years (33.3%, 12/36) compared to those 40 years and older (8.4%, 21/249) (OR 5.43, 95% CI: 2.38–12.38, *p* < 0.0001). Asymmetrical globules or dots were present in 107 cases (37.5%), more frequent in the younger group (55.6%, 20/36) than in the older group (34.9%, 87/249) (OR 2.33, 95% CI: 1.15–4.72, *p* = 0.0192). The globular pattern was identified in five cases (1.75%), significantly more common in patients under 40 years (8.3%, 3/36) compared to older patients (0.8%, 2/249) (OR 11.23, 95% CI: 1.81–69.69, *p* = 0.0094). Similarly, the homogeneous pattern was found in 30 cases (10.5%), with higher prevalence in younger patients (22.2%, 8/36) versus older patients (8.8%, 22/249) (OR 2.95, 95% CI: 1.20–7.25, *p* = 0.0185).

Conversely, scar-like depigmentation was observed in 73 cases (25.6%), more frequent in patients aged 40 years and older (28.5%, 71/249) than in younger patients (5.6%, 2/36) (OR 0.15, 95% CI: 0.04–0.63, *p* = 0.0098). Peppering was present in 102 cases (35.8%), with higher frequency in the older group (38.2%, 95/249) compared to younger patients (19.4%, 7/36) (OR 0.39, 95% CI: 0.17–0.93, *p* = 0.0333). Color asymmetry was observed in 191 cases (67.0%), significantly more common among patients aged 40 years and older (70.3%, 175/249) than in those under 40 (44.4%, 16/36) (OR 0.34, 95% CI: 0.17–0.69, *p* = 0.0028).

**Figure 3:**
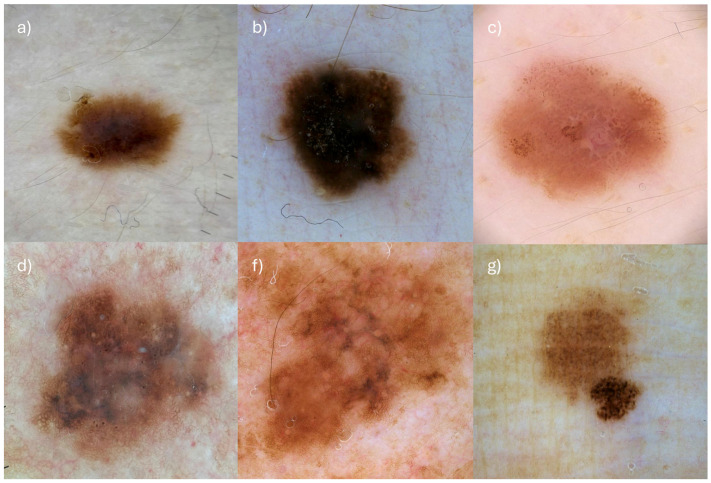
(**a**) melanoma pT1a, Breslow 0.7 mm, in a 28 year-old male presenting as a homogeneous, spitzoid-looking lesion of small diameter (0.5 mm) with irregular dots at the periphery. (**b**) Melanoma in situ in a 35 year-old female presenting as a homogeneous, pigmented lesion with irregular globules at the periphery. (**c**) Melanoma pT1a, Breslow 0.5 mm, in a 16 year-old female presenting as an hypopigmented brown, spitzoid-looking lesion with irregular dots and globules, pseudopods, negative pigment network and irregular vessels. (**d**) Melanoma in situ in a 89-year-old male presenting as a regressive, asymmetrical lesion with scar-like depigmentation, peppering, and blue-gray veil. (**f**) Melanoma in situ in a 75 year-old male presenting as a 1 cm-wide, asymmetrical, regressive lesion with angulated lines, gray color, and peppering. (**g**) Melanoma pT1a, Breslow 0.3 mm, in a 79-year-old female with significant color asymmetry and irregular dots at the periphery.

No statistically significant differences were found between the two age groups for other dermoscopic features. Abrupt ending of pigment network was observed in 106 of 285 cases (37.2%), with similar frequencies in younger patients (13/36, 36.1%) and older patients (93/249, 37.3%) (*p* = 0.891). Atypical blotches were present in 96 cases (33.7%), occurring in 27.8% of younger patients (10/36) and 34.5% of older patients (86/249) (*p* = 0.456). Structureless areas were seen in 241 cases (84.6%), nearly equally distributed between younger (30/36, 83.3%) and older patients (211/249, 84.7%) (*p* = 0.845). Atypical pigment network was reported in 191 cases (67.0%), with 69.4% in the younger group (25/36) and 66.7% in the older group (166/249) (*p* = 0.753). Blue-gray veil was identified in 66 cases (23.2%), present in 22.2% of younger patients (8/36) and 23.3% of older patients (58/249) (*p* = 0.882). Radial streaks occurred in 30 cases (10.5%), with 13.9% prevalence in younger patients (5/36) and 10.0% in older patients (25/249) (*p* = 0.495). Polymorphous vessels were observed in 70 cases (24.6%), slightly less frequent in younger (7/36, 19.4%) than older patients (63/249, 25.3%) (*p* = 0.437). White shiny lines were present in 34 cases (11.9%), with 13.9% in the younger group (5/36) and 11.6% in the older group (29/249) (*p* = 0.671). Angulated lines were noted in 25 cases (8.8%), with similar frequencies in younger (3/36, 8.3%) and older patients (22/249, 8.8%) (*p* = 0.913). A negative pigment network appeared in 22 cases (7.7%), with comparable proportions in younger (3/36, 8.3%) and older patients (19/249, 7.6%) (*p* = 0.865). Prominent skin markings were rare (13 cases, 4.6%), observed in 5.6% of younger patients (2/36) and 4.4% of older patients (11/249) (*p* = 0.727). Asymmetry of pattern was recorded in 189 cases (66.3%), with nearly identical frequencies in younger (24/36, 66.7%) and older patients (165/249, 66.3%) (*p* = 0.953). Gray color was present in 154 cases (54.0%), reported in 58.3% of younger (21/36) and 53.4% of older patients (133/249) (*p* = 0.589). The presence of four or more colors was noted in 144 cases (50.5%), with 55.6% prevalence in younger (20/36) and 49.8% in older patients (124/249) (*p* = 0.524). Reticular pattern was identified in 81 cases (28.4%), distributed similarly between younger (10/36, 27.8%) and older patients (71/249, 28.5%) (*p* = 0.927). Multicomponent pattern was observed in 165 cases (57.9%), with 52.8% in younger (19/36) and 58.6% in older patients (146/249) (*p* = 0.511). Lastly, the non-specific (featureless) pattern was very rare, found in only 2 of 285 cases (0.7%), both in older patients (2/249, 0.8%), with none in younger patients (0/36) (*p* = 1.000). Examples of melanomas from both age groups are shown in Figure 3.

### 3.5. Analysis of Histopathological Parameters

The analysis of histopathological parameters across the 285 cases revealed an overall presence of mitosis of 16.84%, observed in 22.22% (8/36) of patients under 40 years and 16.06% (40/249) of those aged 40 and above; however, the Chi-square test with Yates’ correction showed no statistically significant difference between the two age groups (*p* = 0.494). A quantitative assessment of mitotic figures using the Mann–Whitney U test also confirmed the absence of significant differences (*p* = 0.239), with mean mitotic counts of 5.50 in younger patients and 3.95 in older patients. Ulceration was present in 6.0% of cases (17/285), occurring in 11.1% of patients under 40 and 5.2% of those 40 and older, without a statistically significant difference (*p* = 0.246). Histopathological regression was reported in 18.6% of cases (53/285), more frequently in younger patients (27.8%) compared to older patients (17.3%), yet this difference was not statistically significant (*p* = 0.1986). Finally, solar elastosis was identified in 14.4% of cases (41/285), absent in patients under 40 and present in 16.5% of those aged 40 or older, with a statistically significant difference confirmed by Fisher’s exact test (Table 5, *p* = 0.0041).

### 3.6. Analysis of Histological Subtype

The superficial spreading melanoma (SSM) subtype was identified in 229 out of 285 cases, representing an overall frequency of 80.4%. Among patients younger than 40 years, SSM accounted for 83.3% (30/36) of cases, while in patients aged 40 years or older, it accounted for 79.9% (199/249). Lentigo maligna melanoma (LMM) was observed in 36 out of 285 cases (12.6%), exclusively in patients aged 40 years or older (14.5%, 36/249), with no cases in the younger group. Nodular melanoma (NM) was diagnosed in 14 patients (4.9% overall), representing 11.1% (4/36) of cases in the younger group and 4.0% (10/249) in the older group. Mucosal melanoma and acral lentiginous melanoma (ALM) were each found in 2 cases (0.7%), exclusively among patients aged 40 years or older (0.8%, 2/249 for each subtype), absent in the younger cohort. Spitz melanoma (SM) was observed in 2 cases (0.7%), exclusively in the younger group (5.6%, 2/36), and absent in the older cohort. These findings indicate that SSM is the predominant histological subtype across both age groups, with a slightly higher proportion in the younger patients (83.3%) compared to older patients (79.9%). Conversely, subtypes such as SM were observed exclusively in the younger group, whereas LMM, mucosal melanoma, and ALM were restricted to the older group in this cohort. Inferential analysis using the Chi-square test with Yates’ correction demonstrated a statistically significant difference in subtype distribution between the two age groups (χ**^2^** with Yates’ correction, *p* = 0.000355), indicating a significant association between patient age and melanoma histological subtype (Table 6).

### 3.7. Statistical Analysis of the Association Between Dermoscopic and Histologic Regression

We assessed the association between dermoscopic features of regression—scar-like depigmentation and peppering—and histologic regression in the overall cohort and stratified by age groups. In the overall population, logistic regression demonstrated significant associations for both scar-like depigmentation (*p* = 0.018, OR 2.21, 95% CI: 1.14–4.29) and peppering (*p* = 0.021, OR 2.12, 95% CI: 1.12–4.02) with histologic regression. Among patients younger than 40 years, scar-like depigmentation was not significantly associated with histologic regression (*p* = 1.00), whereas peppering showed a strong and significant association (*p* = 0.015, OR 11.65, 95% CI: 1.62–88.70). In patients aged 40 years or older, scar-like depigmentation was significantly linked to histologic regression (*p* = 0.012, OR 2.47, 95% CI: 1.22–5.03), and peppering also demonstrated a significant association (*p* = 0.010, OR 2.40, 95% CI: 1.23–4.68), suggesting both features are important predictors of histologic regression in this age group.

## 4. Discussion

The incidence of MM is rapidly increasing across Europe, affecting both in situ and invasive forms. This rise is attributed to multiple factors, including a genuine increase in incidence, as well as improved diagnostic accuracy facilitated by advanced non-invasive techniques such as dermoscopy, digital videodermoscopy, total-body photography, and confocal microscopy [19]. Additionally, a certain degree of clinical and pathological overdiagnosis has been reported. A major debate in the scientific literature concerns the actual impact of enhanced diagnostic capabilities on melanoma-related morbidity and mortality [13]. Despite the surge in diagnoses of in situ and minimally invasive melanomas, a proportional decrease in thick melanomas has not been observed. If early-stage melanomas were true precursors to advanced lesions, one would expect a decline in the incidence of thick melanomas. However, epidemiological data indicate that while the detection of thin melanomas has increased significantly, the incidence of thick melanomas has also risen. This paradox raises questions regarding the effectiveness of widespread population-based melanoma screening, shifting the focus towards targeted screening strategies [20]. Current recommendations favor screening individuals at higher risk—those with a personal or family history of melanoma, fair skin, a high nevus count, dysplastic nevus syndrome, or signs of chronic sun damage—rather than indiscriminate population screening [21,22]. Given this perspective, refining risk stratification within the population is crucial to improving diagnostic accuracy and identifying high-risk lesions at an early stage. However, relatively few studies have investigated melanoma characteristics while stratifying patients by age. Currently, data on the clinical, pathological, and prognostic features of MM in individuals under 40 years of age remain inadequate. This gap is partly because melanoma is more prevalent in individuals over 40 [12]. The limited epidemiological studies based on cancer registries suggest that melanoma in younger individuals exhibits distinct epidemiological, clinical, and prognostic features compared to that in middle-aged and older adults. Specifically, in younger patients, melanoma appears to have a female predominance (potentially influenced by estrogenic factors and sun exposure patterns), a predilection for the trunk and lower extremities, a higher frequency of the spitzoid histological subtype, and a relatively better prognosis [2,23,24]. It is important to recognize that our study sample is not fully representative of the general population, as it was derived from a secondary referral dermatology clinic where patients were evaluated for lesions of uncertain interpretation. Despite this limitation, our findings align with existing literature in certain aspects, such as the different anatomical distribution of melanoma between younger and older individuals. In older patients, MM is more frequently observed in chronically sun-exposed areas, such as the face and upper limbs, and exhibits a lentiginous and regressive appearance. To our knowledge, this is the first study to specifically analyze age-related variations in the expression of melanoma-specific dermoscopic criteria. A previous study by Slowinska et al. examined differences in risk factors and general dermoscopic patterns (e.g., multicomponent, spitzoid, amelanotic) across three age groups (<30 years, 30–60 years, >60 years) but did not conduct an in-depth analysis of validated melanoma-specific dermoscopic criteria [25]. Nevertheless, their findings highlighted significant age-related dermoscopic differences, with younger patients (<30 years) more frequently exhibiting spitzoid lesions, while older patients (>60 years) presented with lesions displaying lentiginous and regressive features [25]. Our findings support these observations, showing that younger patients tend to have melanomas with a higher prevalence of growth-associated features (e.g., atypical globules/dots, pseudopods) and a more homogeneous pattern, contributing to a spitzoid appearance. In contrast, older patients more frequently display regressive dermoscopic features (e.g., scar-like depigmentation, peppering), resulting in a high frequency of melanomas with a lentiginous appearance and asymmetry of color. The presence of asymmetrical globules or dots was significantly more frequent in the younger patient group (55.6% vs. 34.9%), consistent with dermoscopic features indicative of tumor growth and proliferation. This finding aligns with the observed higher prevalence of spitzoid and growth-associated patterns in younger individuals. Conversely, peppering was more commonly detected in older patients (38.2% vs. 19.4%), likely reflecting cumulative UV exposure and a more chronic, fibrotic regression process typical of aged skin. A particularly significant finding in our study is the observed difference in mean Breslow thickness between the two age subgroups. Patients under 40 years of age showed a mean Breslow thickness greater than 1 mm, a value significantly higher compared to that observed in the older subgroup. This difference could be partially attributed to a small number of extreme values influencing the mean (as suggested by the coinciding median values in both groups) and to the heterogeneous sample size. A limitation of the present study is the uneven distribution of the sample across age groups, with a substantially smaller number of patients under 40 years compared to the older cohort (36 vs. 249). This imbalance reflects the actual epidemiological prevalence of melanoma, which is less common in younger populations. Nevertheless, the analysis revealed a statistically significant difference in Breslow thickness between the two groups (*p* = 0.011), indicating a genuine difference in the clinical behavior of melanoma in younger patients. These findings support the hypothesis that melanoma in younger individuals may present with distinct biological and clinical characteristics, such as greater thickness at diagnosis, warranting further investigation in larger and more targeted cohorts. However, it also raises the possibility that a subset of biologically aggressive melanomas in younger patients may be more challenging to diagnose due to their distinct dermoscopic presentation, ultimately impacting prognostic factors in this age group. Another relevant finding emerging from our study is the significantly larger lesion size observed in patients aged 40 years or older (mean size = 0.91 cm). This observation, seemingly in contrast with the greater mean Breslow thickness detected in younger patients, may reflect increased clinical detectability due to a more expansive superficial growth pattern. Conversely, melanomas in younger patients appear to exhibit a more aggressive behavior, despite maintaining smaller dimension (mean size = 0.73 cm). In our cohort, the presence of dermoscopic regression—defined according to established criteria in the literature as scar-like depigmentation and peppering—was identified as a significant predictor of histopathological regression. Univariate logistic regression analysis revealed a statistically significant association between scar-like depigmentation and histological regression (*p* = 0.0013), with an OR of 2.8 (95% CI: 1.49–5.22). Similarly, peppering was significantly associated with histological regression (*p* = 0.0018), yielding an OR of 2.6 (95% CI: 1.43–4.83). Taken together, the results indicate that melanomas exhibiting dermoscopic signs of regression have nearly a threefold increased likelihood of concurrent histopathological regression. Importantly, our study confirms the well-established correlation between dermoscopic regression features and their histological counterparts, as previously reported in the literature and supported by daily clinical practice. Moreover, we explored an additional discriminative factor: the age of the patient at the time of melanoma diagnosis. Our results demonstrated a differential predictive value of dermoscopic regression patterns across age groups. Although both peppering and scar-like depigmentation were more frequently observed in patients aged ≥40 years, peppering was significantly associated with histopathologic regression exclusively in younger patients (<40 years), whereas scar-like depigmentation retained predictive value only in the older group (≥40 years). This finding aligns with existing literature, which associates peppering with inflammatory-type regression, characterized histologically by dense lymphocytic infiltrates, dermal melanophages, and reduced epidermal melanocytes—features typical of early, immune-mediated melanoma regression, possibly more active in younger individuals. Conversely, scar-like depigmentation has been linked to fibrotic regression, with histological hallmarks such as dermal fibrosis, epidermal atrophy, and solar elastosis, more commonly seen in chronically sun-exposed skin of older patients [21]. These age-related differences suggest that peppering and scar-like depigmentation may reflect distinct phases or pathways of the regression process, influenced by host immune competence and cumulative UV exposure. This supports the need for an age-aware interpretation of regression features in dermoscopic assessment and melanoma prognostication. [26]. In this context, it is worth highlighting a case–control study by De Giorgi et al. on thin melanomas, which reported that dermoscopic regression may indicate a metastatic potential of the neoplasm [27]. Furthermore, a retrospective analysis by Slowinska et al. investigating clinical, dermoscopic, and histopathological features of melanoma found that these characteristics—particularly those associated with regression—vary significantly with age [25]. Inflammatory regression features, including peppering, were notably more frequent in younger patients. A possible pathophysiological explanation for these age-related differences may lie in the immunological variations across age groups. Younger individuals likely possess a more robust immune response, which could result in acute inflammatory activity against the neoplasm. Clinically, this manifests as peppering; histologically, as dense perilesional infiltrates of lymphocytes and melanophages. In contrast, older patients may exhibit a more attenuated or chronic immune response, promoting fibrotic remodeling and lesion involution, seen clinically as scar-like depigmentation and histologically as late-stage fibrotic regression. A relevant biological factor that may modulate MM development and progression in relation to age is the role of reactive oxygen species. Aging is associated with increased oxidative stress and diminished antioxidant defenses, promoting DNA damage and carcinogenesis [28,29]. Conversely, younger individuals may benefit from more effective immune surveillance and different redox environments, potentially explaining variations in melanoma behavior [30]. Age-stratified logistic regression analysis in our cohort further confirmed the variable predictive performance of dermoscopic markers across age groups. Scar-like depigmentation was not a significant predictor of histological regression in patients under 40 years of age (*p* = 1.00), while the association remained statistically significant in the ≥40 age group (*p* = 0.0016), suggesting that a subset of histological regression in younger individuals may not be captured by dermoscopy. In contrast, peppering retained its predictive value in younger patients (*p* = 0.015) but lost statistical significance in the older patients (*p* = 0.079). To our knowledge, this is the first study to directly correlate dermoscopic regression features with histopathological regression while highlighting the influence of patient age. These findings offer novel insights into the biology of melanocytic regression and underscore the importance of personalized interpretation of regression features in melanoma diagnostics and prognostication. Further large-scale investigations are warranted to validate our preliminary findings and better characterize the melanoma profile in younger patients, a demographic that remains underrepresented in the current literature. We emphasize the importance of educating young individuals on melanoma risk factors (e.g., inadequate sun protection) and encouraging prompt dermatological evaluation in cases of newly developing lesions or changes in pre-existing lesions. We believe that self-monitoring plays a more crucial role in detecting high-risk lesions in this age group than generalized population-based screening. In the future, artificial intelligence and teledermatology may help reduce the current barriers to timely dermatological assessment, ultimately improving early melanoma detection in younger patients, particularly for the rare but clinically deceptive invasive lesions.

## 5. Conclusions

Melanoma-specific dermoscopic features vary according to patient age. In younger individuals, growth-associated features (such as atypical globules/dots and pseudopods) and a homogeneous pattern are more frequently observed, giving the lesion a spitzoid appearance. This finding is supported by the exclusive presence of the Spitz melanoma subtype in this age group. Furthermore, patients under the age of 40 exhibited a significantly higher mean Breslow thickness, suggesting that these lesions may represent biologically more aggressive neoplasms. In contrast, among patients aged 40 years or older, a higher prevalence of regression-associated features—such as scar-like depigmentation and peppering—was detected. This may reflect the cumulative effect of ultraviolet radiation exposure and the differing anatomical distribution of melanomas in older patients, with a greater proportion arising in photo-exposed and chronically sun-damaged areas such as the face. These features are typically indicative of lentiginous lesions with an overall asymmetric dermoscopic pattern. Notably, solar elastosis was observed exclusively in the ≥40 age group, further supporting the link between this histopathologic feature, skin aging, and chronic sun exposure. Patient age also appeared to influence lesion size at diagnosis. Individuals aged 40 years or older presented with clinically more advanced lesions in terms of surface diameter. In our cohort, dermoscopic regression—defined by the presence of scar-like depigmentation and peppering—was identified as a significant predictor of histopathological regression. It is important to highlight that patient age critically modulates this association: scar-like depigmentation retained its predictive value exclusively in patients aged 40 or older, whereas peppering remained a significant predictor only in younger patients. This may indicate that the two dermoscopic features represent distinct phases of the regression process and are associated with differing histopathological changes depending on the patient’s age. Further studies with larger sample sizes are warranted to validate these preliminary findings and to better characterize the under—40 population, which remains insufficiently explored to date.

## Figures and Tables

**Table 1:** Anatomical site of MM in general population.

Site	Number of cases	Percentage (%)
Posterior trunk	90	31.58
Anterior trunk	60	21.05
Lower limbs	46	16.14
Upper limbs	42	14.74
Face	35	12.28
Scalp	6	2.11
Palmoplantar	3	1.05
Others	3	1.05

**Table 2:** Anatomical site of MM in patient under 40 years of age.

Site	Number of cases	Percentage (%)
Posterior trunk	13	30.56
Anterior trunk	11	36.11
Lower limbs	10	27.78
Others	2	5.56

**Table 3:** Anatomical site of MM in patient aged 40 years or older.

Site	Number of cases	Percentage (%)
Posterior trunk	77	30.92
Anterior trunk	49	19.68
Upper limbs	41	16.47
Lower limbs	36	14.46
Face	36	14.46
Scalp	5	2.01
Palmoplantar	3	1.20
Others	2	0.80

**Table 4:** Dermoscopic parameters with statistically significant differences between the two populations (<40 years vs. 40 years and above).

Population	Parameter	OR	95% CI	*p*-Value
≥40 years	Asymmetric globules/dots	0.15	0.035–0.630	**0.0098**
≥40 years	Peppering	0.39	0.165–0.982	**0.0333**
≥40 years	Color asymmetry	0.34	0.166−0.689	**0.0028**
<40 years	Asymmetric globules/dots	2.33	1.148–4.721	**0.0192**
<40 years	Pseudopods	5.43	2.380–12.382	**<** **0.0001**
<40 years	Globular pattern	11.23	1.809–69.689	**0.0094**
<40 years	Homogeneous pattern	2.95	1.199–7.247	**0.0185**

**Table 5:** Significant histologic result.

Parameter		*p*-Value
Solar elastosis		**0.0041**

**Table 6:** Distribution of MM hystological subtypes by age group.

Population	SSM	LMM	NM	Mucosal	ALM	SM
All	229	36	14	2	2	2
<40 years	30	0	4	0	0	2
≥40 years	199	36	10	2	2	0

## Data Availability

The raw data supporting the conclusions of this article will be made available by the authors on request.

## Abbreviations

The following abbreviations are used in this manuscript:

MM	Malignant melanoma
SSM	Superficial spreading melanoma
LMM	Lentigo maligna melanoma
NM	Nodular melanoma
SM	Spitz melanoma
ALM	Acral lentiginous melanoma
